# Accident de travail pas très commun

**DOI:** 10.11604/pamj.2015.22.35.6737

**Published:** 2015-09-16

**Authors:** Redouane Ouakrim, Mohammed Saleh Berrada

**Affiliations:** 1 Service de Chirurgie Orthopédique, CHU Ibn Sina, Université Mohammed V, Rabat, Maroc

**Keywords:** Corps etranger, arthrite, parage, Foreign body, arthritis, trimming

## Image en medicine

Il s'agit d'un patient de 40 ans, admis au service des urgences pour un traumatisme par pistolet pneumatique à clou du membre inferieur droit. Le traitement initial à base d'amoxicilline protégée, parage et ablation du corps étranger étaient efficaces. L'aggravation clinique à une semaine d'intervalle vers un empâtement du genou et douleur à la marche. Une ponction articulaire du genou a retrouvé un liquide louche et des BGN à l'examen direct. Une plaie à coté du genou ou du cul de sac supérieur nécessitent de préférence un lavage articulaire associé au parage.

**Figure 1:**
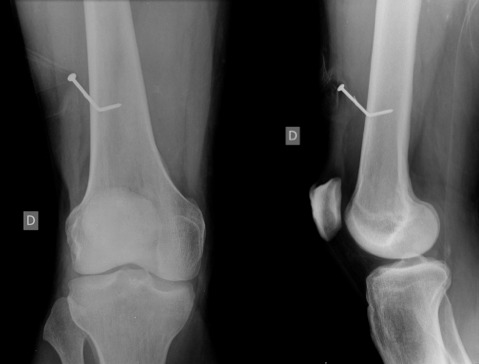
Radiographie du genou montrant un clou intraosseux

